# Complete nutrition drink with retrograded starch is low glycemic, and the individual glucose response to the low glycemic complete nutrition drink depends on fasting insulin levels and HOMA-IR in a randomized cross-over control trial

**DOI:** 10.1017/jns.2022.23

**Published:** 2022-04-01

**Authors:** Warisara Wongniyomkaset, Numphung Rungraung, Niramol Muangpracha, Thunnalin Winuprasith, Dunyaporn Trachootham

**Affiliations:** 1 Master of Science Program in Nutrition and Dietetics, Institute of Nutrition, Mahidol University, Nakhon Pathom 73170, Thailand; 2 Institute of Nutrition, Mahidol University, Nakhon Pathom 73170, Thailand

**Keywords:** Clinical trial, Complete nutrition drink, Glycemic index, Healthy volunteers, Insulin, Personalised nutrition, Postprandial glucose response, Retrograded starch, ALT, alanine aminotransferase, AST, aspartate aminotransferase, AUC_i_, incremental area under the curve, BMI, Body mass index, BUN, blood urea nitrogen, CI, confidence intervals, DM, Diabetes Mellitus, ECLIA, electrochemiluminescence immunoassay, FPG, fasting plasma glucose, GI, Glycemic index, h, hour, HDL, high-density lipoprotein, HDL-C, high-density lipoprotein cholesterol, IPAQ, International physical activity questionnaire, LDL, low-density lipoprotein, MET, metabolic equivalent, min, minute, SD, standard deviation, SE, standard error

## Abstract

Complete nutrition drinks with a low glycemic index (GI) provide nutritional support and prevent hyperglycaemia. The present study identified GI and factors predicting individual glucose response to a new complete nutrition drink. A randomised cross-over controlled trial was conducted in eighteen healthy volunteers (FPG < 100 mg/dl). Complete nutrition drinks containing retrograded starch, glucose solution and white bread were assigned in a random sequence with 14-day wash-out intervals. Plasma glucose and insulin levels were measured from baseline to 180 min after consuming each food. Results show the adjusted GIs of the drink was 48.2 ± 10.4 and 46.7 ± 12.7 with glucose and white bread as the reference, respectively. While the drink has low GI (<55), the individual glucose responses varied (GI: 7–149). Comparing characters in individual GI < 55 (*n =* 12) and GI ≥ 55 (*n =* 6) groups revealed significantly higher baseline insulin in the low GI group (14.86 ± 16.51 μIU/ml *v*. 4.9 ± 3.4 μIU/ml, *P* < 0·05). The correlation matrix confirms only two predictive factors for having individual GI <55 were baseline insulin (*r* = 0·5, *P* = 0·03) and HOMA-IR (*r* = 0·55, *P* = 0·02). ROC curve reveals fasting insulin above 1.6 μIU/ml and HOMA-IR above 1.05 as the cut-off values. The findings suggest that the complete nutrition drink has a low GI, but there was wide variability in individual responses partly explained by fasting insulin levels and HOMA-IR. Screening for fasting insulin and HOMA-IR may be encouraged to maximise the functional benefit of the drink.

## Introduction

Hyperglycaemia, or high blood glucose level, is usually caused by the inability of cells to fully respond to insulin^(1)^. Diet management plays a key role in both diabetes prevention in healthy people and glycemic control in diabetic patients^(2)^. Various factors affect glycemic response including characteristics of the food (e.g. nutrient composition, ripeness, cooking method and processing), as well as the individual characters (e.g. clinical status, physical activity, microbiome profiles)^(2,3)^. Previous studies have shown that glycemic index (GI) is a strong predictor of glycemic response^(4)^. Consumption of food with a high GI may increase the risk of hyperglycaemia and insulin resistance^(5)^.

GI is a ranking system for carbohydrate containing foods based on their postprandial glycemic responses^(5)^. According to the recommendation, foods with GI values of less than 55, 55–70 and more than 70 are classified as low GI, medium GI and high GI, respectively^(6)^. A previous study showed that a low GI diet such as bean puree induced a lower glycemic response than that of a high GI diet such as potato^(7)^. A study in obese pubertal boys reported that low GI food enhanced satiety and lower voluntary intake^(8)^. Importantly, many studies suggested that a low-GI diet helped control blood sugar and reduced the risk of Type II Diabetes Mellitus (DM)^(9–11)^.

Oral nutrition support is an important dietary intervention tool for people with food insecurity, vulnerability and malnutrition risks, such as elderly people, children and patients with chewing and swallowing difficulties^(12,13)^. We formulated a complete nutrition drink in the same way as a enteralnutrition formula in which macronutrients are added for proper energy input and distribution and micronutrients are added to meet the daily nutrition requirement^(14)^. A variety of complete nutrition formulas has been generated over the past decades to serve a functional purpose, for example, low GI, high fibre, high protein, and specialised formula for infants, patients with renal disease or cancer^(14–17)^. Among these functional varieties, a complete nutrition drink with a low GI is one of the most popular since it can also be applied for healthy people with the concern of blood sugar control^(14,16,18)^. Interestingly, a previous clinical trial showed that a liquid form of food has a lower GI than that of a solid form of food with equal energy density and protein content^(19)^.

Previous studies showed that complete nutrition drinks with a low GI could provide nutritional support and prevent hyperglycaemia^(18)^. Meanwhile, a previous study in 800 people showed that different people have a tremendously varied glycemic response to the same meal^(3)^. Consistently, another clinical study observed a wide variation up to 5 fold of glycemic response in different persons towards the same meal (glucose, sports drink and brown rice syrup)^(20)^. The individual GIs of the same food could be in different categories of GI (low vs high)^(20)^. Usually, the calculation of the GI is based on the average value of area under the blood glucose/time curves between the test food and the reference food of several people. Therefore, the conclusion that a kind of food has a low GI does not guarantee that all people will have a low glycemic response towards such a diet^(20)^.

In the present study, a complete nutrition formula aiming to reduce postprandial glycemic response was formulated with retrograded starch and other basic nutrients. A previous *in vitro* study showed that retrogradation could reduce starch digestibility and estimated GI values for all types of rice cultivars^(21)^. However, the clinical GI values of food products made by retrogradation were unknown. This randomised control trial was conducted to identify the GI of the complete nutrition drink. The primary findings of the present study suggest that the average GI value of the complete nutrition drink placed it in the low GI category. The secondary outcome showed that baseline insulin and HOMA-IR may be the significant predictive factors for responding to complete nutrition drinks.

## Materials and methods

### Ethical aspects and setting

The protocol MU-CIRB 2018/148.2007 was approved by the Mahidol University Central Institutional Review Board (MU-CIRB) with the COA. No. MU-CIRB 2018/163.1109. This research was performed according to the International Council for Harmonization of Technical Requirements for Pharmaceuticals for Human Use Good Clinical Practice (ICH-GCP) and the Declaration of Helsinki. The informed written consent was obtained from each participant before the study. The protocol was registered in the Thai Clinical Trial Registry (TCTR20210305001). The protocol can be accessed at http://www.thaiclinicaltrials.org/.

### Study design, blinding, random allocation and concealment

A randomised cross-over controlled trial was used. Participants who passed the screening were randomly assigned into three groups. Minimisation was applied to ensure that all groups were matched for sex, age, body mass index (BMI) and biochemical data. Each group received a different sequence of test foods, i.e. complete nutrition drink, glucose solution, and white bread. A researcher performed random allocation. Sample collectors and laboratory analyzers were blinded from the test food until the end of the study.

### Participants

The inclusion criteria for screening participants were healthy people aged more than 18 years old who had BMI less than 30 kg/m^2^, no systematic diseases and normal blood biochemical parameters (complete blood count, aspartate aminotransferase (AST), alanine aminotransferase (ALT), bilirubin, blood urea nitrogen (BUN), creatinine, cholesterol, high-density lipoprotein (HDL), low-density lipoprotein (LDL), triacylglycerols and fasting plasma glucose (FPG)). The exclusion criteria of participants included alcoholism, cigarette smoking, pregnancy, dairy or gluten allergy, under medical therapy and unable to maintain regular physical activity throughout the study. All participants signed their informed written consent before data collection.

### Sample size and power

Previous studies utilised fifteen participants for GI identification of starchy food^(22)^. However, the present study required three visits of data collection and each visit required multiple venous blood collections. Considering the expected withdrawal of the participants, 20 % drop-out, the sample size was set at eighteen (*n =* 18). *Post-hoc* power analysis was performed to confirm the adequacy of the sample size. The effect size of 0.428 was obtained from data of primary outcome measure (area under the curve incremental (AUC_i_)). Then, *post-hoc* power was calculated with an α-error value of 0·05 for *F* tests-ANOVA repeated measures between factors and total measurements of 54 (18 subjects × 3 measurements). The *post-hoc* power for the primary outcome measure, area under the curve incremental (AUC_i_) was 0.928. The *post hoc* power for secondary outcome measure, factors predicting GI < 55, was 0.84. Power analyses wereperformed by using G-power V.3.1.9.2.

### Intervention and materials

The participants consumed 250 ml of complete nutrition drink, 75 g of white bread, and 250 ml of glucose solution. Each test food contained 35 g of available carbohydrate equally as recommended (25–50 g of available carbohydrate^(5)^). Since complete nutrition drink has a thick liquid consistency, we utilised both white bread and glucose solution as the reference foods^(23)^. Complete nutrition drink was made by dissolving the powder in warm water (around 65°C). The powder was made from retrograded starch and other nutrients. It was manufactured by Chiangmai Bioveggie Co., Ltd. White bread was made from wheat flour, sucrose, vegetable fat, yeast, blends, milk powder and iodised salt. The participants were asked to consume 250 ml of water with white bread^(5)^. White bread was a product of President Bakery Public Company, Ltd. (Bangkok, Thailand). Glucose solution was made by dissolving 35 g glucose powder (Utopian, Co., Ltd., Thailand) in 250 ml of drinking water. Table 1 shows nutrient contents of 250 ml of complete nutrition drink, 35 g of glucose solution and 75 g of white bread. All of these food items had an equal amount of carbohydrates.

**Table 1.** Nutrient contents of complete nutrition drink (250 mL), glucose solution (35 g) and white bread (75 g)

	Complete nutrition drink	Glucose solution	White bread
Total energy (kcal)	280	140	203
Energy from fat (kcal)	60	0	31.3
Total fat (g)	7	ND	3·9
Saturated fat (g)	4	ND	2.3
Cholesterol (mg)	20	ND	ND
Protein (g)	13	ND	7.8
Total carbohydrate (g)	41	35	35
Digestible carbohydrate (g)	36	35	33
Dietary fibre (g)	5	ND	2
Sugar (g)	6	35	4.7
Vitamin B-1 (mg)	0.68	ND	0.1
Vitamin B-2 (mg)	0.99	ND	0.1
Vitamin B-6 (mg)	0.91	ND	ND
Vitamin B-12 (μg)	2.74	ND	ND
Sodium (mg)	310	ND	343.7
Calcium (mg)	679	ND	25
Iron (mg)	4.71	ND	0.5

ND, not detectable.

### Study procedure

The study was conducted at the Institute of Nutrition, Mahidol University. Participants who passed the screening were randomly assigned into three groups. The first, second and third groups were started with complete nutrition drinks, glucose solution and white bread, respectively. Each intervention was separated by 14 days wash-out periods. After overnight fasting, participants consumed each food within 5 min. They were instructed to sit comfortably. Venous blood samples were collected at 0, 5, 20, 35, 65, 95, 125, 155 and 185 min of each test day, i.e. at fasting (before food intake), at 0, 15, 30, 60, 90, 120, 150 and 180 min, respectively, after finishing the food for determining the plasma glucose level^(24)^. All participants finished the food within 5 min. Thus, the time point of blood collection at 5 min was the time immediately (at 0 min) after consuming the food. Plasma insulin was measured at 0 min (baseline) and postprandial intervals of 30, 45, 60, 90, 120, 150 and 180 min.

### Monitoring

The habitual diet and physical activity were assessed by using dietary records and an international physical activity questionnaire (IPAQ) during wash-out periods^(25)^. In each wash-out period, all participants were asked to do 3-day food records weekly which included two weekdays and one weekend day. Energy and dietary intake were analysed by using the INMUCAL-Nutrient V.3.2 program^(25)^. Habitual physical activity was divided into low, medium and high levels according to their total MET-minute-week of physical activities^(26,27)^. Before the test day, participants were asked to refrain from food for 12–15 h. Sipping water was allowed. All participants consume similar last evening meals (rice stir-fried fried vegetables and meat) before all test days^(28)^. Furthermore, all participants were asked to avoid heavy exercise, abstain from heavy meals at least 24 h before the test, abstain from supplements and drugs that affect digestion and glucose metabolism, abstain from alcoholic beverages and tobacco smoking throughout the study^(25)^.

### Clinical outcome measurement

The primary outcome measures were postprandial glucose response and GI of complete nutrition drink. The plasma glucose level was measured by an enzymatic hexokinase method^(25)^ using Rayto RT-9200 (Rayto Life and Analytical Sciences Co., Ltd., Shenzhen, P.R. China). A line graph between plasma glucose concentration and time was generated for each participant for each test food. AUC_i_ of glucose for each test food was calculated geometrically over 3 h^(4)^. Only the values above fasting concentration were used to compute AUC_i_^(29)^. GI values were calculated by using both glucose and white bread as references. The following equation was used for the calculation of GI using glucose as a reference^(5)^.
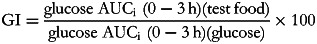


The average GI of white bread was 71.2, compared to 100 for glucose solution. Thus, when using white bread as a reference, the GI must be divided by 1.4 as follows^(24,30)^.
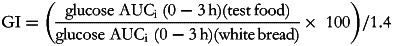


The secondary outcome measure was plasma insulin response. Plasma insulin concentration was measured by electrochemiluminescence immunoassay (ECLIA)^(31)^ using cobas® 8000 modular analyzer series (Roche Roche Diagnostics International AG, Rotkreuz, Switzerland). A line graph between plasma insulin concentration and time was generated for each participant for each test food. AUC_i_ of insulin for each test food was calculated geometrically over a 3 h statistics.

### Statistics

The baseline numerical characteristics of participants were displayed using mean and standard deviation (sd). Statistical tests were selected based on the normality of data. Normality tests were performed for all data by using the Shapiro–Wilk test. The comparison of baseline characteristics among three randomised groups was analysed by one-way ANOVA for normally distributed data or Kruskal–Wallis test for skewed distributed data. The average plasma glucose levels at each time point were compared among different foods by using Repeated measures ANOVA followed by Tukey's tests. Baseline glucose levels and AUC_i_ of glucose or insulin were compared among different test foods by using the Friedman test followed by Dunn's test. Bonferroni correction was applied if multiple comparisons are performed. Physical activity levels and dietary intakes were compared between the two wash-out periods by using the *χ*^2^ test and Wilcoxon signed-rank test, respectively. Secondary exploratory analysis was performed to identify key factors affecting glycemic response. Spearman rank correlation analyses were performed to identify the relationship between having individual GI <55 and multiple variables including baseline characteristics (age, sex, BMI), Laboratory data (baseline insulin, fasting glucose, HOMA-IR, HbA1c, BUN, Creatinine, eGFR, Cholesterol, triacylglycerols, HDL, LDL, HDL-c ratio, AST, ALT, Total bilirubin) as well as dietary intake, and physical activity. ROC curve analysis was performed to identify if the variable was a good predictor of having individual low GI. All statistical tests were performed by using a two-tailed test. *P*-value < 0·05 was considered statistically significant. Graph Pad Prism V.9.0.2 was used for graphing and statistical analysis.

## Results

### Participant flowchart

**Fig. 1.**
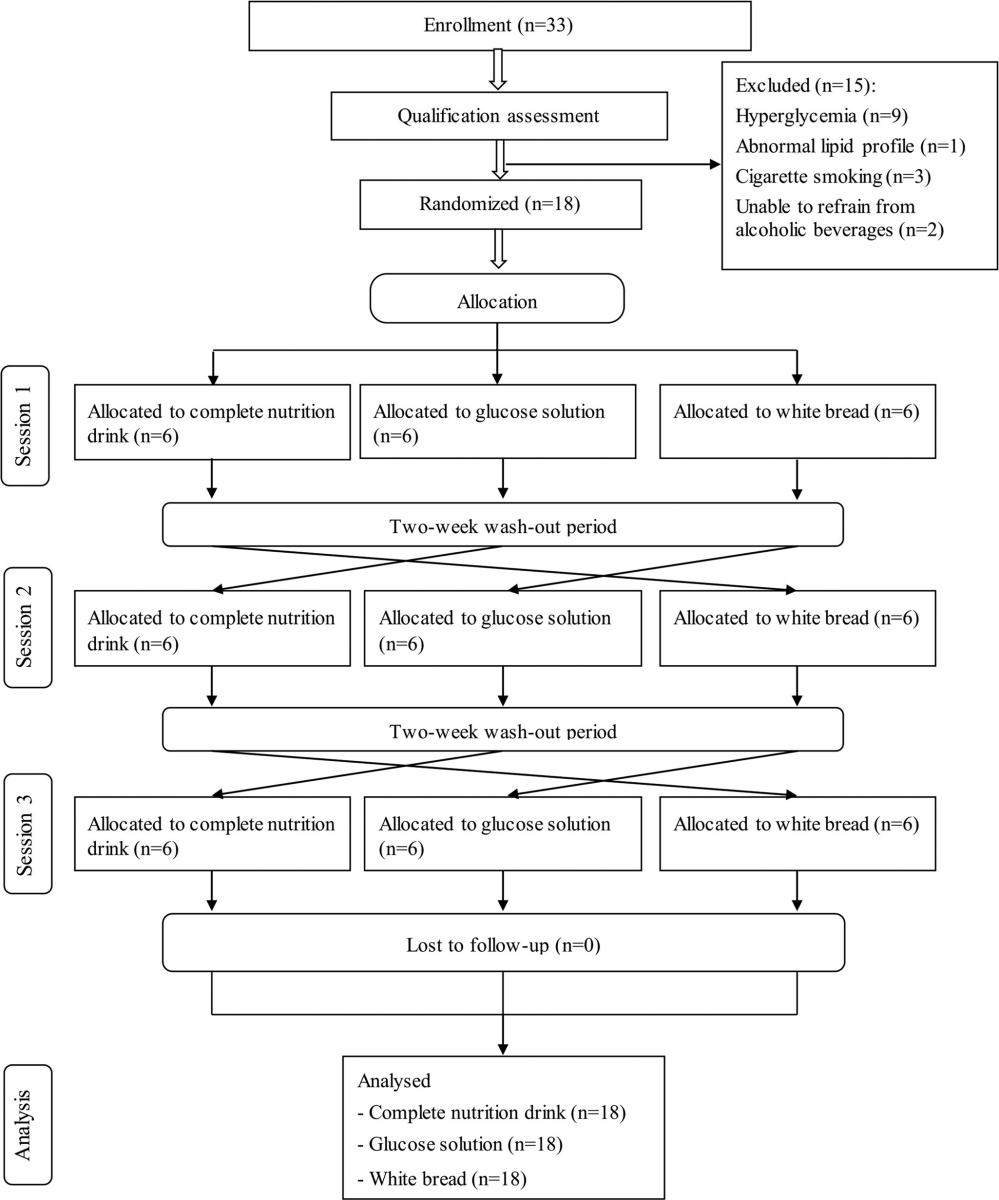
CONSORT participants’ flowchart.

The present study was conducted from June to September 2020. Fig. 1 shows the Consolidated Standards of Reporting Trials (CONSORT) diagram. Thirty-three volunteers were recruited to the study. After the screening, fifteen subjects were excluded. Eighteen participants including nine males and nine females (22–48 years) were randomly assigned into three groups. All participants completed all tests; thus, the data were intention-to-treat analysed from all randomised participants.

### Baseline characteristics

As shown in Table 2, all average baseline laboratory parameters, except for total cholesterol, were in the normal range. Although the mean of total cholesterol was higher than the normal range, the means of total cholesterol to HDL-C ratios were normal (less than 5·0 for males and less than 4·5 for females^(32)^). Supplementary Table S1 shows no statistically significant differences in age, BMI and laboratory characteristics among the randomised groups (*P*-value ≥ 0·05).

**Table 2.** Demographic characteristics of all participants (*n* 18)

Characteristics	Value		Reference range
	Mean	sd	
Socio-demographic data			
Male: Female (*n*)	9: 9		
Age, years	31.78	8.06	
Anthropometry data			
BMI, kg/m^2^	21.12^a^	2.15	
Biochemical data			
Fasting plasma glucose, mg/dl	95.22	5.94	<100
HbA1c, %	5.2	0.4	4.8−5.7
BUN, mg/dl	11.17	2.94	6−20
Creatinine, mg/dl	0.77	0.14	0.5−1.5
Total cholesterol, mg/dl	221.1	31.35	<200
Triacylglycerols, mg/dl	98.11	30.73	<150
HDL-C, mg/dl	62.78	12.6	>45
Male	58.89	13.98	>45
Female	66.67	10.39	>55
LDL-C, mg/dl	138.7	29.03	<130
Total cholesterol: HDL-C ratio	3.3	0.63	
Male	3.45	0.77	<5.0
Female	3.14	0.45	<4.5
AST, U/l	13	4.49	0−40
ALT, U/l	17	8.44	0−40
Total bilirubin, mg/dl	0.57	0.21	0−1.2

BMI, body mass index; BUN, blood urea nitrogen; HDL-C, high-density lipoprotein cholesterol; LDL, low-density lipoprotein cholesterol; Total cholesterol: HDL-C ratio, total cholesterol divide by HDL-C; AST, aspartate transaminase; ALT, alanine transaminase; U/L: unit per litre.

a BMI of participants was in the range of 18.1–24.8 kg/m^2^.

### Postprandial glucose response

**Fig. 2.**
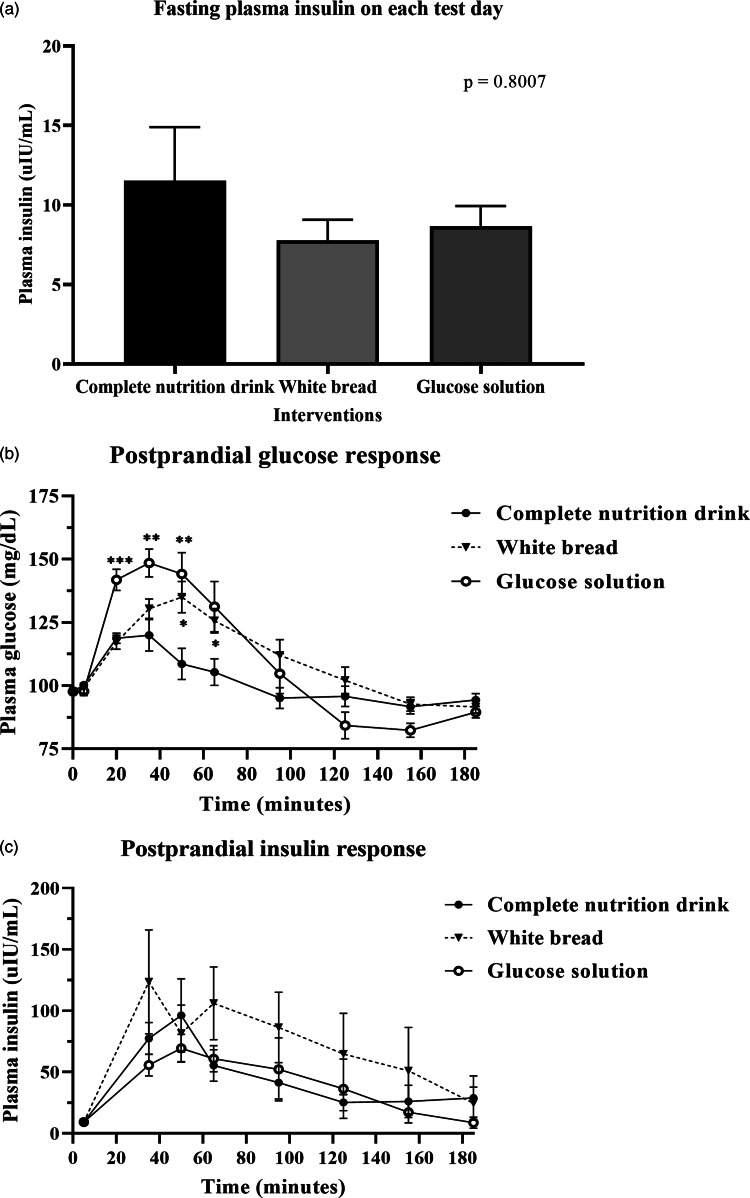
Postprandial glucose and insulin response after test food consumption. (a) Bar graph showed mean ± sem of plasma insulin concentration (mg/dl) in all participants (*n* 18) at baseline before complete nutrition drink, white bread and glucose solution consumption as specified. The *P*-value was from the Friedman test. (b) mean ± sem of plasma glucose concentration (mg/dl) in all participants (*n* 18) at 0, 5, 20, 35, 65, 95, 125, 155 and 185 min of each test day, i.e. fasting (before food intake), at 0, 15, 30, 60, 90, 120, 150 and 180 min, respectively, after complete nutrition drink, white bread and glucose solution consumption as specified. (*) means *P*-value < 0·05, (**) means *P*-value < 0·01, (***) means *P*-value < 0·001, repeated measures two-way ANOVA followed by Tukey's multiple comparison tests. The *P*-value for the interaction time-by-treatment was *P* < 0·0001. (c) mean ± se plasma insulin concentration (μIU/ml) in all participants (*n* 18) at 0, 30, 60, 90, 120, 150 and 180 min after complete nutrition drink, white bread and glucose solution consumption as specified. *P*-value was obtained from repeated measures two-way ANOVA.

After consuming the complete nutrition drink, the postprandial glucose concentration rapidly increased and peaked at 20 min. In contrast, those of glucose solution and white bread were reached maximum levels at 35 and 50 min, respectively (Fig. 2(b)). While the plasma glucose level of complete nutrition drink was declined first, those of glucose solution and white bread continuously remained high. Table 3 shows that the average AUC_i_ of glucose response for complete nutrition drink (mean ± se: 1574 ± 378·0; 95 % CI 833·5, 2420) was significantly lower than those of glucose solution (*P* = 0·0026; mean ± se: 3612 ± 577·9; 95 % CI 2393, 4831) and white bread (*P* = 0·0001; mean ± se: 2974 ± 448·6, 95 % CI 2028, 3921). The average GI of complete nutrition drink was 48·2 ± 10·4 when using glucose solution as the reference food, which was not statistically different from the GI calculated when using white bread as the reference food (46·7 ± 12·7; *P* > 0·99).

**Table 3.** Individual and average area under curve incremental (AUC_i_) of postprandial glucose response and glycemic index (GI) values of complete nutrition drink

Subject code	AUC_i_ CND^a^	AUC_i_ Bread	AUC_i_ GS^b^	GI CND/Bread^c^	GI CND/GS^d^
1a	1168.0	1725.0	5357.0	48	22
1b	303.1	2028.0	3182.0	11	10
1c	4151.0	1238.0	4641.0	239	89
1d	842.5	4485.0	891.9	13	94
1e	222.0	2340.0	1469.0	7	15
1f	1836.0	6174.0	6474.0	21	28
2a	2465.0	2112.0	8529.0	83	29
2b	2264.0	2745.0	4350.0	59	52
2c	131.1	174.9	1368.0	54	10
2d	5679.0	5734.0	6779.0	71	84
2e	4103.0	7436.0	2286.0	39	179
2f	156.8	3744.0	865.9	3	18
3a	1096.0	1968.0	1510.0	40	73
3b	189.8	3115.0	2537.0	4	7
3c	1146.0	1388.0	2670.0	59	43
3d	622.4	3474.0	905.7	13	69
3e	526.4	1769.0	7465.0	21	7
3f	1436.0	1888.0	3734.0	54	38
Mean	**1574**	**2974**	**3612**	**46·7**	**48·2**
se	**378**	**449**	**578**	**12·7**	**10·4**
*P*-value		**<0.01^e^**	**<0.0001^e^**		**>0.99^f^**
CV	**2.55**	**1.47**	**1.37**	**15.74**	**13.79**

a AUC_i_ CND: area under curve incremental of glucose and time after consuming complete nutrition drink.

b AUC_i_ GS: area under curve incremental of glucose and time after consuming glucose solution.

c GI CND/Bread: glycemic index calculated from the ratios of AUC_i_ between that of complete nutrition drink and bread divided by conversion factor of 1.4.

d GI CND/GS: glycemic index calculated from the ratios of AUC_i_ between that of complete nutrition drink and glucose solution.

e *P*-values were from comparing AUC_i_ Bread or AUC_i_ GS with that of AUC_i_ CND by Friedman test followed by Dunn's multiple comparison test.

f *P*-value was from comparing GI CND/GS with that of GI CND/Bread by Wilcoxon matched-pairs signed-rank test. CV, the inter-individual coefficient of variation.

### Postprandial insulin response

Fig. 2(a) shows no significant difference in fasting insulin levels among all test days. Fig. 2(c) shows the postprandial insulin concentration increased and peaked at 50 min after consuming a complete nutrition drink, while the highest peak for glucose solution and white bread were at 50 and 35 min, respectively. The plasma insulin responses of complete nutrition drink continuously remained higher than baseline throughout the 3-h period. In contrast, the insulin response to glucose solution and white bread were rapidly declined. Nevertheless, there were no statistically significant differences among groups. Supplementary Table S2 shows that the average AUC_i_ of insulin response for complete nutrition drink (mean ± se: 6317 ± 1788; 95 % CI 2544, 10 089) was higher than that of glucose solution (mean ± se: 5710 ± 1880; 95 % CI 1743, 9677) but lower than that of white bread (mean ± se: 11 378 ± 4690; 95 % CI 1483, 21 274). However, no statistically significant differences were observed among groups. The average maximum insulin concentrations of complete nutrition drink, white bread and glucose solution were 96·19, 123·51 and 69·5 μIU/ml, respectively.

### Factors affecting individual glycemic response and GI

**Fig. 3.**
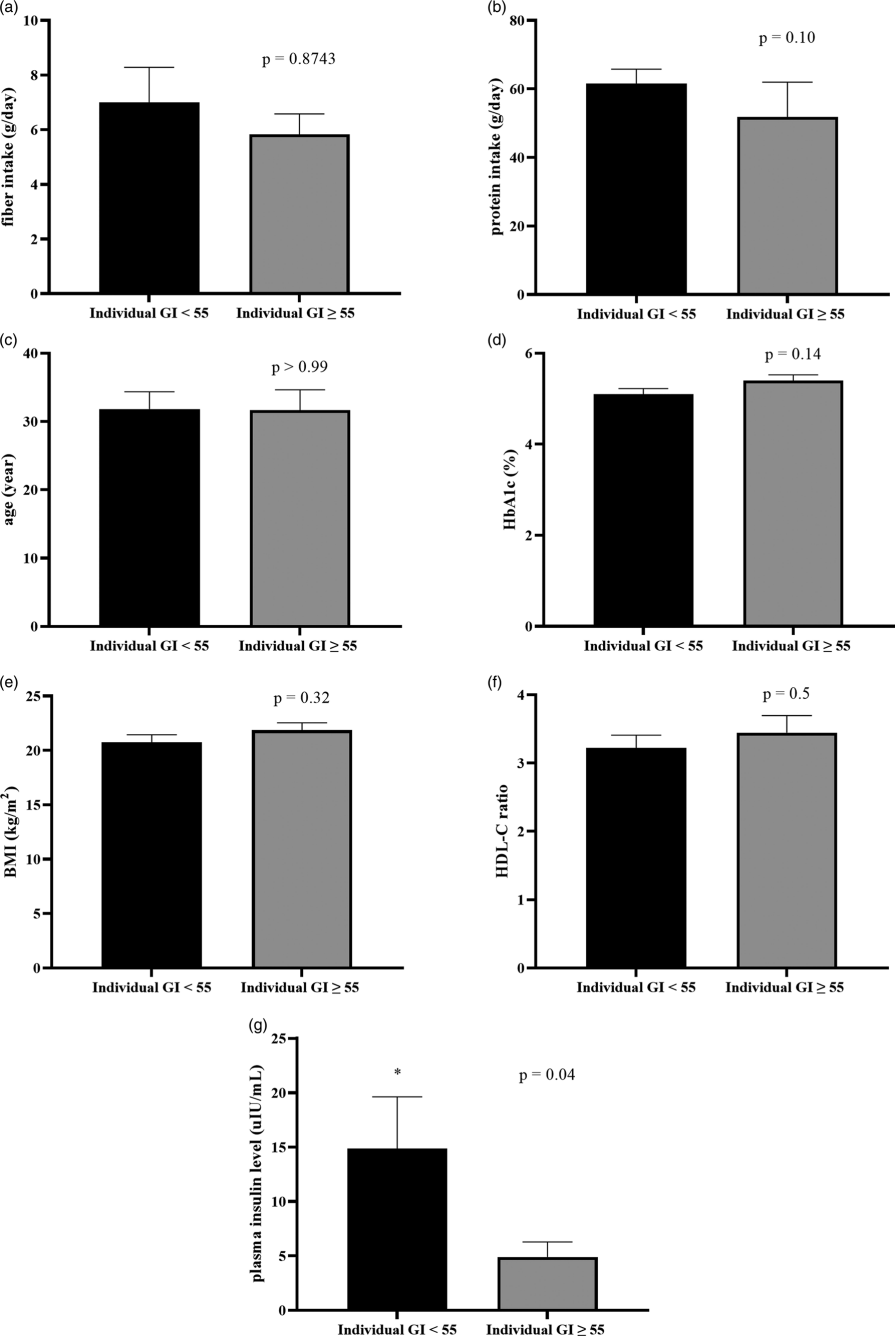
The comparison of baseline characteristics between individual GI <55 and individual GI ≥ 55 groups, when using glucose solution as the reference food. Bar graph showed a comparison of mean ± se of fibre intake (a: g/day), protein intake (b: g/day), age (c: year), HbA1c (d: %), BMI (e: kg/m^2^), HDL-C ratio (f) and baseline insulin (g: μIU/ml) of individual GI <55 and individual GI ≥ 55 groups. *P*-values were obtained from Mann–Whitney tests except for HbA1C, BMI and HDL-C ratio which were from unpaired *t*-tests.

**Fig. 4.**
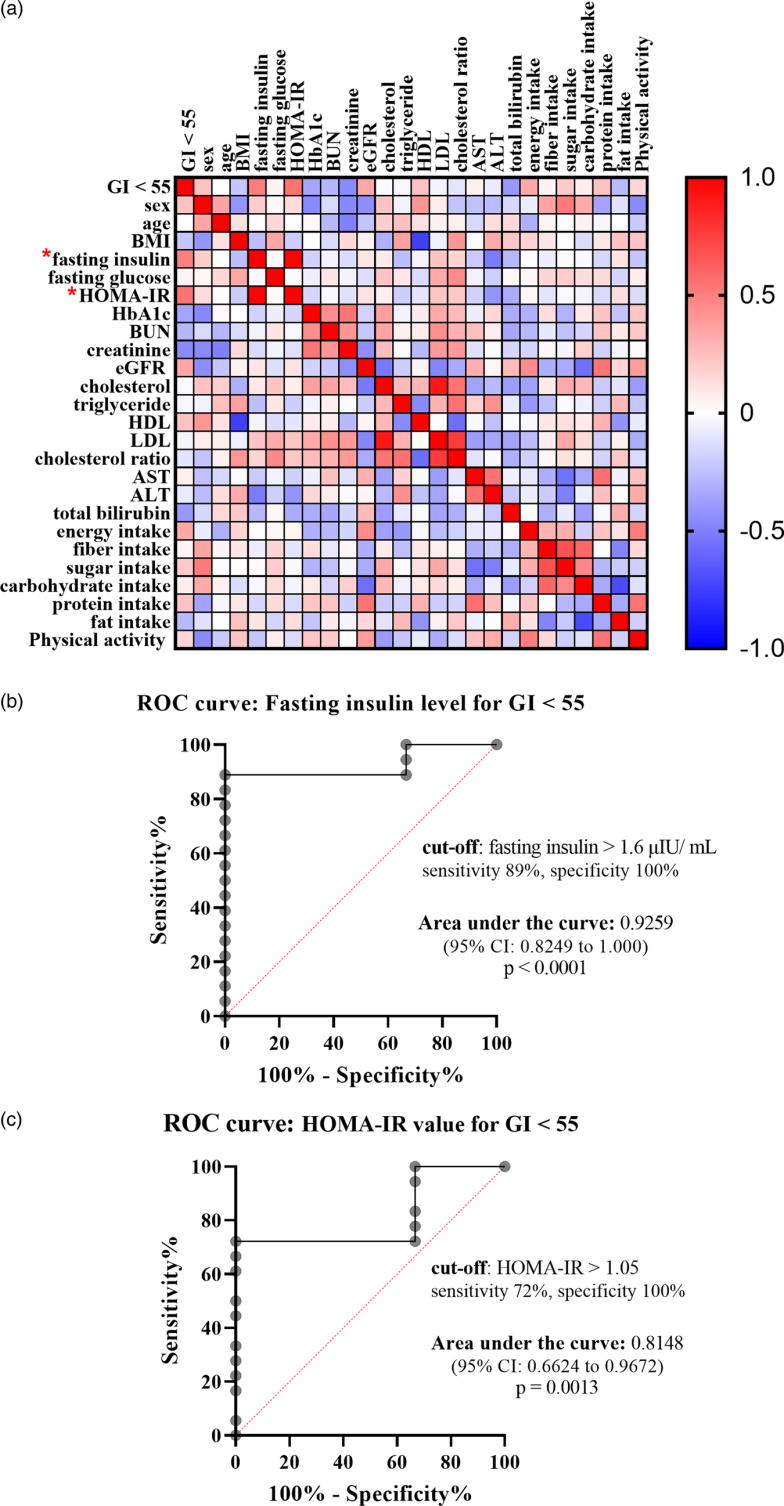
Correlation between having individual GI <55 and various factors. (a) Heat map showed correlation coefficients between specified factors, analysed by Spearman rank correlation analysis. The red and blue boxes indicated positive and negative correlation, respectively. (*) means *P*-value < 0·05. (b and c) ROC curve showed sensitivity and specificity of fasting insulin levels (b) and HOMA-IR (c) in predicting individual GI <55. The area under the ROC curve and the optimum cut-off level were shown.

To identify what factors could predict individual glycemic response to the complete nutrition drink with an average low GI, individual GI was used for the analysis. The individual GI was calculated as the percentage of the AUC_i_ of complete nutrition drink compared to the glucose of each participant. The individual GI <55 groups included the participants showing low GI of the complete nutrition drink, while the individual GI ≥ 55 showed medium or high GI. Supplementary Table S3 shows the list of the participants in the individual GI <55 and ≥55 groups. Participants with individual GI <55 were distributed in all three randomised groups suggesting that the sequence of intervention did not affect the response. About the factors affecting response to the low-GI complete nutrition drink, baseline characteristics and dietary intakes were compared between Individual GI <55 and Individual GI ≥ 55 groups. Interestingly, baseline plasma insulin level was the only parameter showing a difference between groups. The average baseline insulin levels in the Individual GI <55 group was 14·86 ± 16·51 μIU/ml, which was significantly higher than that of the Individual GI ≥55 group (4·893 ± 3·424 μIU/ml). In contrast, there were no statistically significant differences for other factors between Individual GI <55 and Individual GI ≥55 groups (Fig. 3). Consistently, Fig. 4(a) and Table 4 show that fasting insulin and HOMA-IR were the only two parameters significantly correlated with having individual GI <55 (*r* = 0·4997, *P* = 0·0347, 95 % CI 0·02793, 0·7895 and *r* = 0·5463, *P* = 0·0190, 95 % CI 0·09181, 0·8124, respectively). Other parameter such as FPG was not correlated (*r* = 0·0456, *P* = 0·8574, 95 % CI −0·4425, 0·5129) (Table 4). As shown in Fig. 4(b) and (c), the ROC curve confirmed that fasting insulin and HOMA-IR were good predictors of having low individual GI (AUC: 0·92, *P* < 0·0001 and AUC: 0·815, *P* = 0·0013). As shown in Supplementary Table S4, the fasting insulin above 1·6 μIU/ml was identified as the cut-off with the sensitivity of 89 % and the specificity of 100 %. Moreover, the HOMA-IR above 1·05 was identified as the cut-off was the sensitivity of 72 % and specificity of 100 %. Correlation analysis between individual GI values and multiple variables was also performed. Although BMI was identified as the only parameter having a significant correlation with individual GI value, it was not a good predictor evidenced by a low and non-significant area under the ROC curve (Supplementary Fig. S1).

**Table 4.** Spearman's rank correlation coefficient between having a low glycemic response and various baseline characteristics

Variable	Correlation coefficient (*r*)	95 % CI	*P*-value	*P*-value summary
Sex	0.24	−0·2736 to 0·6418	0.3464	ns
Age (year)	0.00	−0·4785 to 0·4785	>0.9999	ns
BMI (kg/m^2^)	−0.23	−0·6365 to 0·2820	0.3647	ns
Baseline insulin (μIU/ml)	0·50	0·2793 to 0·7895	0.0347	*
Baseline glucose (mg/dl)	0·05	−0·4425 to 0·5129	0.8574	ns
HOMA-IR	0·5463	0·09181 to 0·8124	0.0190	*
HbA1c (%)	−0·3543	−0·7121 to 0·1495	0.1492	ns
BUN (mg/dl)	−0·29	−0·6739 to 0·2205	0.2457	ns
Creatinine (mg/dl)	−0·46	−0·7706 to 0·02026	0.0532	ns
eGFR (ml/min/1·73 m^2^)	0.34	−0·1640 to 0·7047	0.1658	ns
Cholesterol (mg/dl)	−0·02	−0·4958 to 0·4608	0.9287	ns
Triacylglycerols (mg/dl)	−0.03	−0·5044 to 0·4518	0.8931	ns
HDL (mg/dl)	0.24	−0·2702 to 0·6440	0.339	ns
LDL (mg/dl)	−0.05	−0·5128 to 0·4427	0.8579	ns
Total cholesterol/HDL-C	−0.10	−0·5537 to 0·3956	0.6863	ns
AST (U/l)	0.06	−0·4332 to 0·5213	0.822	ns
ALT (U/l)	−0.08	−0·5377 to 0·4146	0.7533	ns
Total bilirubin (mg/dl)	−0.40	−0·7400 to 0·09127	0.0956	ns
Dietary intake				
Energy (kcal)	0.34	−0·1646 to 0·7044	0.1665	ns
Fibre (g)	0.05	−0·4422 to 0·5132	0.8559	ns
Sugar (g)	0.20	−0·3036 to 0·6222	0.4153	ns
CHO distribution (%)	0.07	−0·4238 to 0·5297	0.7868	ns
PRO distribution (%)	0.24	−0·2682 to 0·6453	0.3348	ns
Fat distribution (%)	−0.28	−0·6666 to 0·2331	0.2673	ns
IPAQ score	0.16	−0·3458 to 0·5924	0.5286	ns

The table reports the Spearman's rank coefficient correlation (*r*) for the relationship between each person factor and low-GI response analysed by non-parametric Spearman correlation. Dummy code for outcome (low-GI response): 0 = negative, 1 = positive.

CI, confidence interval; ns, not statistically significant.

* means *P* < 0·05.

### Dietary intake and physical activity

Supplementary Fig. S2 shows that there were no statistically significant differences in energy, protein dietary intake, the percentage of energy distribution from carbohydrates and physical activity levels between two wash-out periods (*P*-value ≥ 0·05).

## Discussion

The complete nutrition drink was categorised as a low GI food according to the standard GI determination methodology. Compelling scientific evidence supports the use of low GI diets for blood glucose control in diabetes and the prevention of obesity^(7,33)^. However, GI values are derived from the average calculation and individual glycemic responses to the same diets are varied^(3,20,30)^. Thus, the benefit of a low glycemic diet will be maximised if it has been given to the right person. Complete nutrition drink with a low GI provides energy and nutrients with an additional benefit for blood sugar control^(18)^. However, what factors can predict the individual response to it are unknown. In the present study, we discovered that baseline insulin and HOMA-IR were the only two factors affecting the response to low glycemic complete nutrition drinks. Two components influencing *in vivo* insulin secretion include basal insulin at the fasting state and the effect from the meal^(34)^. The average baseline insulin of the Individual GI ≥55 group was 4.89 ± 3.4, while the normal range of fasting insulin for a healthy population is between 5 and 15 μIU/ml^(35)^. Therefore, the fasting insulin of the Individual GI ≥55 groups in the present study was slightly lower than the normal reference range. Interestingly, insufficiency of available plasma insulin was shown to link with defects in cellular glucose uptake^(36)^. For this reason, lower insulin concentration at fasting state in Individual GI ≥55 group may contribute to higher AUC_i_ of plasma glucose when test food was ingested. Based on the findings of the present study, individuals with adequate baseline insulin levels could be the optimum target group of the low glycemic complete nutrition drink. A future large-scale study is warranted to confirm such a hypothesis. The present study was conducted in healthy normal weight volunteers (BMI between 18.1 and 24.8 kg/m^2^). Thus, the generalizability of the findings to other populations such as pre-diabetics or obese requires future investigation.

The underlying mechanism behind the low GI value of the complete nutrition drink was likely derived from retrograded rice flour. Retrogradation of starch was shown to increase slowly digestible fractions of carbohydrates^(37,38)^. Such action could reduce postprandial glucose response^(37,38)^. Besides retrograded starch, a complete nutrition drink also contains 19 % protein and 22 % fat which could increase insulin production and slow down glucose absorption^(39,40)^. Moreover, the addition of protein and fat to pure starch decreased the ratio of rapidly starch fraction and increased the sum of slowly starch fraction and a resistant starch fraction^(41)^. This could be explained by native proteins forming a network over the starch granules. Such event results in a significant decrease in the granular degradation caused by amyloglucosidase and α-amylase^(42)^. Moreover, protein can bind on the starch surface and encircle the starch granule which interrupts the starch hydrolysis^(41)^. Another mechanism is the effect of fat on the enzymatic susceptibility of starch hydrolysis. The process of complete nutrition drink production may cause amylose-lipid complexes (ALCs), which occur when fat was added to starch and heat-processed in excess water by extrusion cooking or steam-jet cooking. ALCs were classified as resistant starch types III^(43)^. A previous study reported a decrease in digestibility after 120 min for starch–lipid samples compared to starch without lipids. The decreased hydrolysis of starch–lipid samples can be accounted for the formation of starch–lipid complex, which is more resistant to amylase digestion^(44)^. Such a mechanism could also contribute to the low GI nature of the complete nutrition drink.

After consuming complete nutrition drinks, the peak and AUC_i_ of glycemic response and insulin response were different from glucose solution. In particular, the peak and AUC_i_ of glycemic response were lower than those of glucose solution and the peak and AUC_i_ of insulin response were higher than those of glucose solution. Although the difference in insulin response was not statistically significant. A previous study estimated that variability in insulin responses was derived from the glycemic response (23 %) and macronutrient content (10 %)^(45)^. Therefore, both retrograded starch, protein and fat contents may contribute to the differential glycemic response and insulin response of the complete nutrition drink. Nevertheless, there was wide variability in individual glycemic responses towards the drink (not all people showed low glycemic response). Therefore, a further exploratory analysis was undertaken to elucidate the key personal characteristics responsible for such variation. The finding that baseline insulin levels (above 1·6 μIU/ml) and HOMA-IR (above 1·05) can predict low glycemic response is useful for screening the right person who can maximally benefit from the low-GI complete nutrition drink.

Previous studies showed that factors affecting glycemic response included FPG, insulin levels, smoking and physical activity^(46–48)^. However, in the present study, the response to low glycemic complete nutrition drink was correlated only to the fasting insulin levels and HOMA-IR but not to the FPG or other factors. It is worth noting that in the present study all participants were healthy volunteers with normal fasting glucose (lower than 100 mg/dl). A previous study showed that FPG will influence glycemic response only when HbA1C is increased^(48)^. Furthermore, diabetic patients and healthy volunteers were shown to respond differently when consuming the same meal^(49)^. Taken together, it is likely that the role of fasting blood glucose as a predictive factor for glycemic response may be more prominent in diabetic patients than those in healthy volunteers. Previous studies showed that smoking and sedentary behaviour could result in a high glycemic response^(46)^. However, all participants in the present study were non-smokers with mostly moderate physical activity. Therefore, it is possible that in a healthy population the fasting insulin levels play a more important role than other factors in predicting the glycemic response to a low GI diet. Consistently, a previous study showed that women with gestational diabetes who had normal or higher insulin levels improved their glycemic status better than those with low insulin levels, after receiving education to consume low GI diets^(50)^. ROC curve analysis showed that HOMA-IR can be a good predictor of GI values with a sensitivity of 72 % and specificity of 100 %. Compared with HOMA-IR, fasting insulin is still the better predictor with a sensitivity of 89 % and specificity of 100 %. HOMA-IR is an indirect measurement of insulin resistance^(51)^. In the present study, we did not directly measure glucose tolerance to study insulin resistance. Insulin resistance was shown to be strongly associated with increased BMI and obesity^(52)^. In the present study, participants had BMI between 18·1 and 24·8 kg/m^2^, which was a relatively normal weight. Nevertheless, there is still the possibility that some participants may have insulin resistance associated with other factors such as body fat or body fat and triacylglycerols to high-density lipoprotein cholesterol ratio (TG/HDL-C)^(53)^.

The strength of the present study was the design of a randomised cross-over controlled trial. The sequence of interventions was randomly assigned for match groups. And all participants served as their controls. Such design helps reduce biases from individual metabolisms and residual effects from previous interventions. However, there were some limitations of the present study. First, the present study utilised a small sample size (*n* 18) to identify the cut-off level of fasting insulin (>1·6 μIU/ml) associated with individual low glycemic responses. Future large-scale studies are required to verify the finding. Second, the present study recruited healthy volunteers regardless of physical activity levels. Therefore, their baseline physical activity levels were varied from low to high resulting in a variation in glucose and insulin response among participants. Nevertheless, there was no significant correlation between physical activity levels and having a GI <55. Third, modest sample size, narrow age and BMI ranges of participants precluded the effect of those factors on individualised glycemic response to complete nutrition drink. Thus, future studies with more diverse characters of participants are required to verify the finding of this work. Furthermore, the designed sample size in this work was based on standard GI methodology guidelines. *Post hoc* power of 0·928 was calculated based on the primary outcome measure (AUC_i_). Nevertheless, statistical Type I errors in other data such as insulin or HOMA-IR as the predictive factors of glycemic response is still possible. Further large-scale studies are needed to confirm that insulin was the real best predictor, and the present study can be used for future sample size calculation. Fourth, some other variables affecting postprandial glucose response such as gut microbiome, inflammatory markers such as CRP level were not assessed in the present study^(3)^. Therefore, future research covering these factors was needed to verify the findings of the present study. In addition, GI methodology cannot provide a guide to the relative insulin response for some types of food even though the GI was the one of significant prediction of insulin response^(45)^. Hence, capturing the amount of insulin released in response to a particular food by insulin index (II) would provide more comprehensive metabolic information in response to the test drink. Last, the present study focused on the acute effect of complete nutrition drink and postprandial glucose response. Thus, whether fasting insulin level could affect long-term response to complete nutrition drink remains to be elucidated. Based on the low GI of complete nutrition drink, it will be worthwhile to further investigate its long-term effects on blood glucose control in diabetic patients.

## Conclusion

The findings of the present study suggested that the complete nutrition drink with retrograded starch is a low glycemic product according to the mean GI value. However, the individual glycemic response to the drink depends on adequate fasting insulin levels. Screening for fasting insulin (>1·6 μIU/ml) and HOMA-IR (>1·05) may be encouraged to maximise the functional benefit of the drink.

## Supplementary material

For supplementary material accompanying this paper visit https://doi.org/10.1017/jns.2022.23. click here to view supplementary material
