# Supplementary material for: Complete nutrition drink with retrograded starch is low glycemic, and the individual glucose response to the low glycemic complete nutrition drink depends on fasting insulin levels and HOMA-IR in a randomized cross-over control trial
Source: J Nutr Sci. 2022 Apr 1;11:e25. doi: 10.1017/jns.2022.23 (PMC9003636; doi:10.1017/jns.2022.23)
Supplement: Supplementary file 1 [file S2048679022000234sup001.pdf]

Supplemental data

Table S1: Demographic characteristic of randomized groups

**Characteristics**	**Group 1 (n = 6)**		**Group 2 (n = 6)**		**Group 3 (n = 6)**		**P-value**
	**Mean**	**SD**	**Mean**	**SD**	**Mean**	**SD**	
**Sociodemographic data**							
Male: Female (n)	3: 3		3: 3		3: 3		
Age (years)[a]	30.8	8.93	33.5	7.34	31	9.03	0.83 [b]
**Anthropometry data** [a]							
BMI, kg/m^2	21.2	2	21.4	2.57	20.8	2.22	0.89 [b]
**Biochemical data** [a]							
Fasting plasma glucose, mg/dL	93.5	6.38	98.2	6.59	94	4.47	0.52 [c]
HbA1c, %	5.13	0.43	5.22	0.53	5.25	0.29	0.89 [b]
BUN, mg/dL	11.7	3.56	11	3.16	10.8	2.48	0.95 [c]
Creatinine, mg/dL	0.77	0.08	0.77	0.23	0.78	0.08	0.67 [c]
Total cholesterol, mg/dL	224	35.1	211	23.8	229	36.4	0.59 [b]
Triglyceride, mg/dL	103	43.5	105	30.4	87.2	13.2	0.59 [b]
HDL-C, mg/dL	64.7	16.7	59.3	11.8	64.3	10	0.74 [b]
LDL-C, mg/dL	138	34.7	131	23.2	147	30.8	0.63 [b]
Total cholesterol: HDL-C ratio	3.27	0.73	3.3	0.71	3.32	0.57	0.99
AST, U/L	14.8	5.42	13.5	4.64	10.7	2.58	0.27 [b]
ALT, U/L	17.5	10	18.2	10	15.3	5.96	0.85 [b]
Total bilirubin, mg/dL	0.62	0.21	0.55	0.14	0.53	0.29	0.79 [b]

[a] Values of the parameters were mean ± SD; P-values were from [b] One-way ANOVA, [c] Kruskal-Wallis test; BMI: Body mass index; FPG: Fasting plasma glucose; HDL-C: High density lipoprotein cholesterol; LDL: low-density lipoprotein cholesterol; BUN: Blood urea nitrogen; AST: Aspartate transaminase; ALT: Alanine transaminase; U/L: unit per liter

Table S2: Individual and average area under curve incremental (AUCi) of postprandial insulin response after consuming each test food

Subject No.	AUCi CND [a]	AUCi Bread	AUCi GS [b]
1a	4000	6221	11877
1b	1921	590.8	2913
1c	16084	20136	4201
1d	2236	1515	908.7
1e	2340	84449	5356
1f	5679	32888	5515
2a	5579	2485	5693
2b	8780	13404	35907
2c	0.5349	3597	2873
2d	5890	5838	5646
2e	28198	3052	2868
2f	579.7	5302	1804
3a	6508	2724	627
3b	19485	7371	4187
3c	1284	2269	2677
3d	2798	4657	644.8
3e	0.0001827	6195	5429
3f	2337	2115	3656
**Mean**	**6,317**	**11,378**	**5,710**
**SE**	**1788**	**4690**	**1880**
**P-value [c]**		**0.59**	**0.96**

[a]AUCi CND: area under curve incremental of insulin and time after consuming complete nutrition drink; [b] AUCi GS: area under curve incremental of insulin and time after consuming glucose solution; [c] P-values were obtained from repeated measures ANOVA and Tukey's multiple comparison test, comparing with complete nutrition drink

Table S3: The list of responders for complete nutrition drink

	Glucose solution		White bread	
	Individual GI < 55 (n=12)	Individual GI ≥ 55 (n=6)	Individual GI < 55 (n=13)	Individual GI ≥ 55 (n=5)
subject code	1a	1c	1a	1c
	1b	1d	1b	2a
	1e	2d	1d	2b
	1f	2e	1e	2d
	2a	3a	1f	3c
	2b	3d	2c	
	2c		2e	
	2f		2f	
	3b		3a	
	3c		3b	
	3e		3d	
	3f		3e	
			3f	

The table lists codes of participants who showed low glycemic index of complete nutrition drink (responder), compared to glucose solution or white bread as specified. The code 1, 2, 3 represents the randomized group 1, 2, 3 who received all interventions in different sequences.

Table S4: ROC-curve analysis for prediction of GI ≤ 55 based on fasting insulin level

Cut-off level	Sensitivity%	95% CI	Specificity%	95% CI	Likelihood ratio
> 0.4050	100	82.41% to 100.0%	33.33	16.28% to 56.25%	1.5
> 0.8500	94.44	74.24% to 99.72%	33.33	16.28% to 56.25%	1.417
> 0.9450	88.89	67.20% to 98.03%	33.33	16.28% to 56.25%	1.333
**> 1.600**	**88.89**	**67.20% to 98.03%**	**100**	**82.41% to 100.0%**	
> 2.685	83.33	60.78% to 94.16%	100	82.41% to 100.0%	
> 3.200	77.78	54.79% to 91.00%	100	82.41% to 100.0%	
> 4.175	72.22	49.13% to 87.50%	100	82.41% to 100.0%	
> 5.250	66.67	43.75% to 83.72%	100	82.41% to 100.0%	
> 6.885	61.11	38.62% to 79.69%	100	82.41% to 100.0%	
> 8.545	55.56	33.72% to 75.44%	100	82.41% to 100.0%	
> 8.870	50	29.03% to 70.97%	100	82.41% to 100.0%	
> 9.970	44.44	24.56% to 66.28%	100	82.41% to 100.0%	
> 11.15	38.89	20.31% to 61.38%	100	82.41% to 100.0%	
> 12.15	33.33	16.28% to 56.25%	100	82.41% to 100.0%	
> 12.95	27.78	12.50% to 50.87%	100	82.41% to 100.0%	
> 13.30	22.22	9.001% to 45.21%	100	82.41% to 100.0%	
> 15.30	16.67	5.837% to 39.22%	100	82.41% to 100.0%	
> 17.10	11.11	1.974% to 32.80%	100	82.41% to 100.0%	
> 40.95	5.556	0.2850% to 25.76%	100	82.41% to 100.0%	

Table S5: ROC-curve analysis for prediction of GI ≤ 55 based on HOMA-IR value

Cut-off level	Sensitivity%	95% CI	Specificity%	95% CI	Likelihood ratio
> 0.05000	100	82.41% to 100.0%	33.33	16.28% to 56.25%	1.5
> 0.1500	94.44	74.24% to 99.72%	33.33	16.28% to 56.25%	1.417
> 0.4500	83.33	60.78% to 94.16%	33.33	16.28% to 56.25%	1.25
> 0.7500	77.78	54.79% to 91.00%	33.33	16.28% to 56.25%	1.167
> 0.9000	72.22	49.13% to 87.50%	33.33	16.28% to 56.25%	1.083
**> 1.050**	**72.22**	**49.13% to 87.50%**	**100**	**82.41% to 100.0%**	
> 1.200	66.67	43.75% to 83.72%	100	82.41% to 100.0%	
> 1.650	61.11	38.62% to 79.69%	100	82.41% to 100.0%	
> 2.100	50	29.03% to 70.97%	100	82.41% to 100.0%	
> 2.450	44.44	24.56% to 66.28%	100	82.41% to 100.0%	
> 2.900	33.33	16.28% to 56.25%	100	82.41% to 100.0%	
> 3.150	27.78	12.50% to 50.87%	100	82.41% to 100.0%	
> 3.300	22.22	9.001% to 45.21%	100	82.41% to 100.0%	
> 3.700	16.67	5.837% to 39.22%	100	82.41% to 100.0%	
> 9.750	5.556	0.2850% to 25.76%	100	82.41% to 100.0%	

Figure S1

a

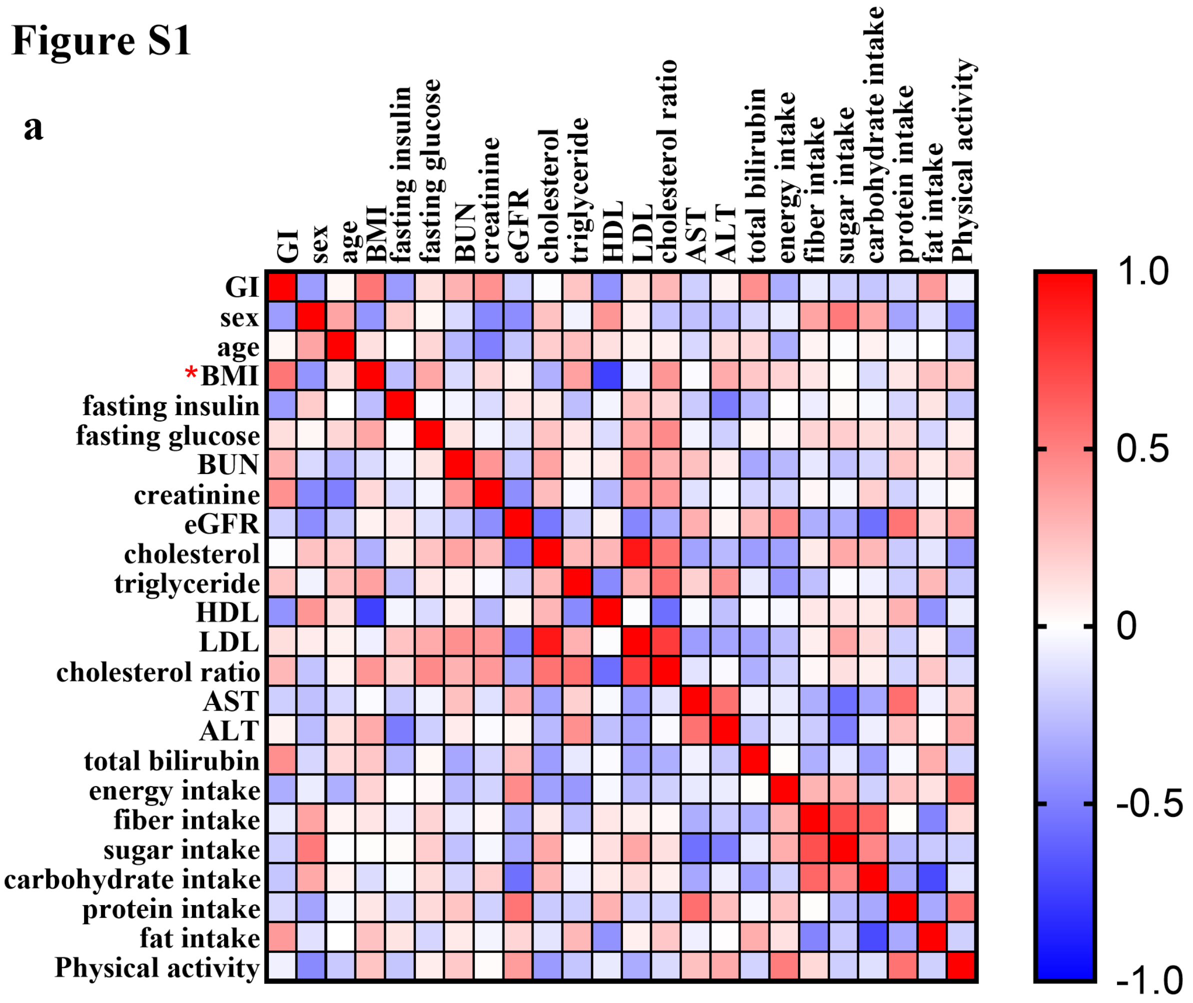
GI
sex
age
*BMI
fasting insulin
fasting glucose
BUN
creatinine
eGFR
cholesterol
triglyceride
HDL
LDL
cholesterol ratio
AST
ALT
total bilirubin
energy intake
fiber intake
sugar intake
carbohydrate intake
protein intake
fat intake
Physical activity
1.0
0.5
0
-0.5
-1.0

b

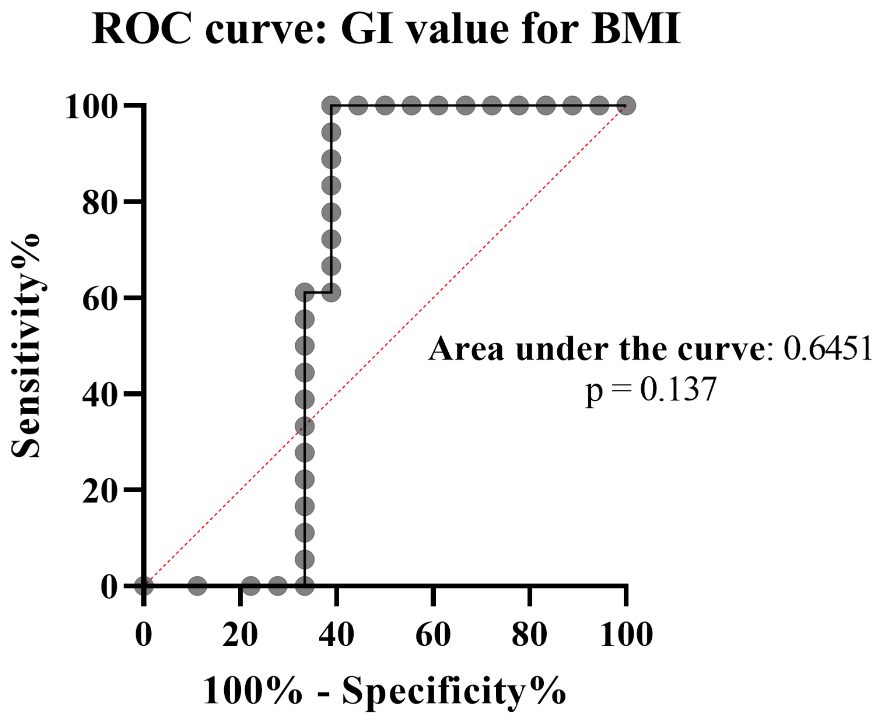
ROC curve: GI value for BMI
Sensitivity%
100% - Specificity%
0
20
40
60
80
100
Area under the curve: 0.6451
p = 0.137

Figure S2

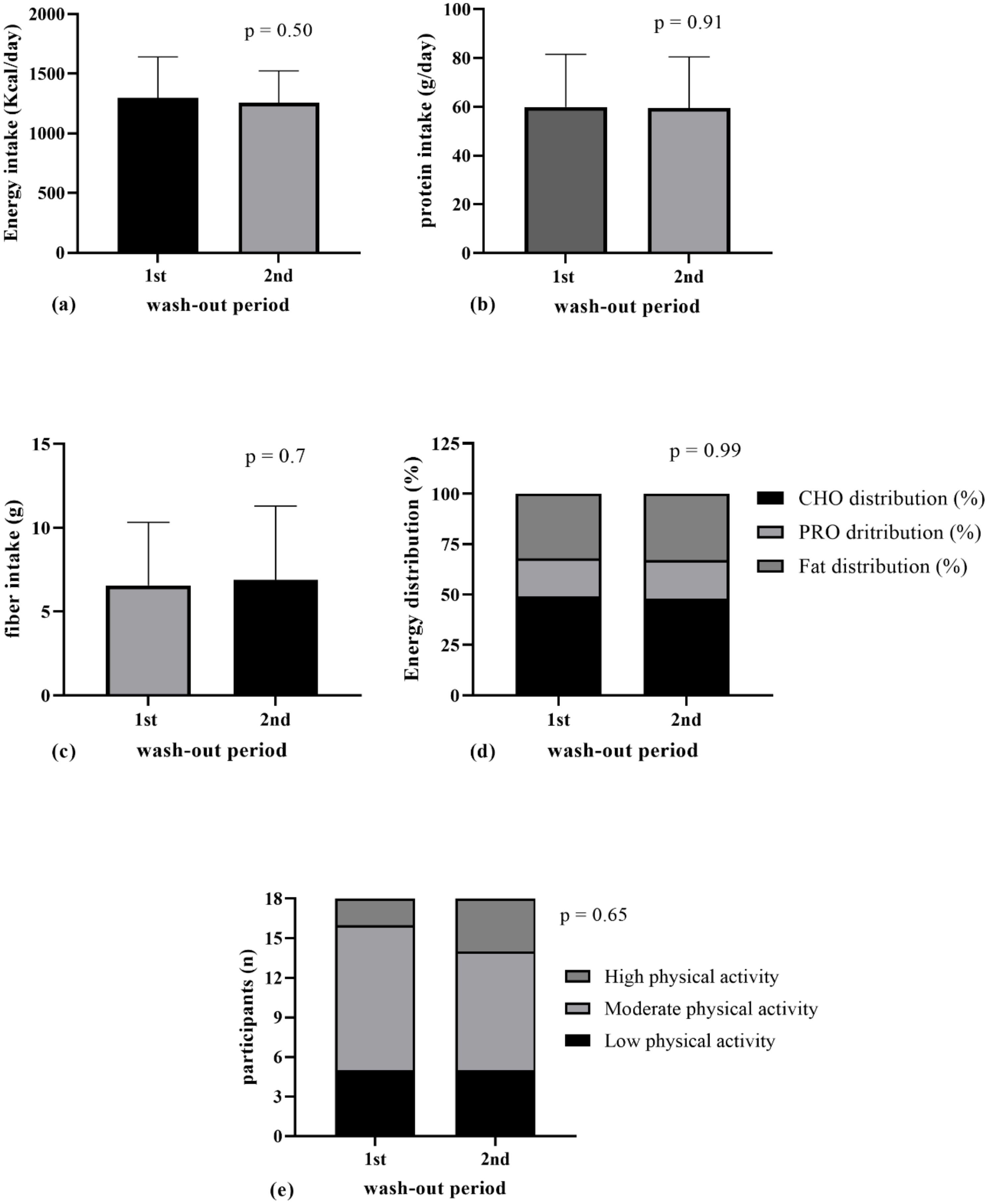

Energy intake (Kcal/day)
2000
1500
1000
500
0
p = 0.50
1st
2nd
(a)
wash-out period
protein intake (g/day)
100
80
60
40
20
0
p = 0.91
1st
2nd
(b)
wash-out period
fiber intake (g)
15
10
5
0
p = 0.7
1st
2nd
(c)
wash-out period
Energy distribution (%)
125
100
75
50
25
0
p = 0.99
CHO distribution (%)
PRO dritribution (%)
Fat distribution (%)
1st
2nd
(d)
wash-out period
participants (n)
18
15
12
9
6
3
0
p = 0.65
High physical activity
Moderate physical activity
Low physical activity
1st
2nd
(e)
wash-out period
